# Detecting depression in speech using verbal behavior analysis: a cross-cultural study

**DOI:** 10.3389/fpsyg.2025.1514918

**Published:** 2025-05-29

**Authors:** Terry Amorese, Marialucia Cuciniello, Claudia Greco, Olga Sheveleva, Gennaro Cordasco, Cornelius Glackin, Gavin McConvey, Zoraida Callejas, Anna Esposito

**Affiliations:** ^1^Department of Psychology, Università degli Studi della Campania “L. Vanvitelli”, Caserta, Italy; ^2^Department of Brain and Behavioural Sciences, Università degli Studi di Pavia, Pavia, Italy; ^3^Intelligent Voice Ltd, London, United Kingdom; ^4^Virtus Caritatis Ltd, Downpatrick, United Kingdom; ^5^Department of Languages and Computer Systems, University of Granada, Granada, Spain

**Keywords:** verbal behavior analysis, depression, language analysis, linguistic content, cross-cultural differences

## Abstract

**Introduction:**

Language analysis has proven to be a reliable methodology for discriminating depressed people from healthy subjects; the present study investigates differences in the linguistic content of spoken interactions from depressed and healthy subjects belonging to three different European areas: Northern Ireland, Italy, and Russia.

**Method:**

The speech of 241 participants (65 native English speakers, 108 native Italian speakers, and 68 native Russian speakers) was analyzed, using the computerized text analysis tool LIWC (Linguistic Inquiry Word Count).

**Results:**

In line with the current literature, it was observed that depressed subjects tended to use more first-person singular pronouns, speak less, use a more negative tone while speaking and use more words related to negative emotions and anxiety compared to healthy controls. Our study also highlighted some innovative findings, such as depressed subjects' greater spontaneity and tendency to speak with less self-censorship compared to healthy participants, as well as a tendency to adopt a type of thinking defined as “informal” rather than analytic. Moreover, our study is the first, at least to the best of our knowledge, comparing speech content of depressed participants belonging to three European areas: Western Europe (Northern Ireland), Southern Europe (Italy) and Eastern Europe (Russia).

**Conclusions:**

Data collected through the present study could be useful in providing guidelines for the design of autonomous systems able to detect early signs of mood changes and depression through the analysis of interactional exchanges. The final aim is to provide automated and cost-effective technological interventions to be used in health care centers, as well as by mental health professionals, such as psychologists, psychiatrists, psychotherapists, therefore with the aim to provide assistance, jointly with the clinician's expertise, in the process of diagnosing depression.

## 1 Introduction

The World Health Organization (2023) estimated that depression affects 3.8% of the world's population, ~280 million people live with depression, making it one of the most common illnesses worldwide. Depression is characterized both by physical symptoms, such as changes in body weight, sleep patterns and psychomotor changes, and by purely psychological and emotional symptoms, such as depressed mood, decreased interest in all activities, feelings of worthlessness, reduced ability to concentrate, and recurring thoughts of death (American Psychiatric Association, 2013). A crucial issue concerns a prompt and accurate diagnosis of depression; to do so a promising methodology is represented by the analysis of language, bearing in mind that depression affects the way people think and communicate. Language and textual analysis, considering linguistic attributes, such as grammar, syntax, and the vocabulary of depressed subjects, have proven to be accurate, reliable, and objective methods, able to discriminate depressed people from healthy subjects (Nguyen et al., 2014; Smirnova et al., 2018; Esposito et al., 2020) as also showed by studies using machine learning techniques for differentiating subjects leaving with depression from healthy control by analyzing speech transcripts (Rohanian et al., 2019; Ilias and Askounis, 2024; Alsenani et al., 2024; Li et al., 2023).

### 1.1 Related work

Previous research on depression linguistic style highlighted that depressed subjects use significantly more first-person singular pronouns (Ramirez-Esparza et al., 2008), use more negative emotion words (Rude et al., 2004), more cognitive mechanism words (Pennebaker et al., 2003), and past tenses (Holman and Silver, 1998). Moreover, it has been observed that depressed people tend to use more absolutist words (Al-Mosaiwi and Johnstone, 2018) and show a decreased verbal output (Bagby et al., 2000); it has also been observed (Capecelatro et al., 2013) that individuals with a long-term history of depression use fewer appetitive words, namely words related to positive emotion, sex, and food. In a recent study (Tlachac and Rundensteiner, 2020) text messages and tweets of people living with depression were analyzed, highlighting that individuals with depression tend to have, compared to controls, a decreased usage of words in certain word categories such as air travel, leadership, real estate, competing, and exercise. A noteworthy topic is represented by cross-cultural differences in the speech patterns of individuals experiencing depression, acknowledging that the cultural context affects both the experience and expression of depressive symptoms. Cultural beliefs significantly shape community attitudes toward mental health and influence treatment methods (Chentsova-Dutton and Tsai, 2009; Ng et al., 2016).

By exploring these variations, the research aims to deepen our understanding of how cultural factors impact the manifestation of depression and perceptions of mental health across different communities. The current findings (De Choudhury et al., 2017) highlight distinctions among depressed individuals from Western (United States, United Kingdom) and Non-Western (South Africa, India) countries. It was found that people with depression from non-western backgrounds generally show more positive emotions and fewer negative emotions than individuals from western cultures. Non-western cultural groups exhibit more references to cognitive processes, certainty terms, and perceptions than western cultural groups. Non-western groups were less inclined to engage in conversations about taboo subjects like religion, death, and sexuality. Western societies tend to focus on topics such as social isolation, death, and self-destructive behavior, while non-western societies were more likely to address the shame associated with experiencing mental illness and share personal struggles related to mental health.

The present study investigates cross-cultural differences in linguistic patterns among Northern Ireland (Western Europe), Italy (Southern Europe), and Russia (Eastern Europe). These cultural contexts vary in their attitudes toward mental health and emotional expression. Western cultures tend to emphasize individualism and direct communication, which may be reflected in a higher use of self-referential language and explicit emotional expression in their linguistic patterns (Hofstede, 2001). Southern European cultures, such as the Italian culture, are often characterized by strong family ties and expressive communication styles, potentially leading to more social-oriented language and a focus on emotional expression (Triandis, 2018). Eastern European cultures, like Russian, may exhibit more collectivistic tendencies and potentially greater emotional restraint, which could manifest in less explicit emotional language and a focus on shared experiences (Markus and Kitayama, 2014). We hypothesize that these cultural differences will be reflected in the linguistic patterns of individuals with depression, with variations observed in self-referential language, emotional expression, and social orientation across the three groups.

### 1.2 Our contributions

The present work started in the context of a research project called “Androids” (AutoNomous DiscoveRy of Depressive Disorder Signs) which was aimed at developing an automatic depression diagnosis support system. The aim is to identify features of speech expressions that may signal the presence of a depressive state. This work is based on verbal behavior analysis, which allows the investigation of both the content (what is said, i.e., nouns) and the linguistic style (how it is said, e.g., pronouns, prepositions, articles; Tausczik and Pennebaker, 2010); two aspects which could reflect cognitive patterns (e.g., self-focus, pessimism, low self-esteem; Beck et al., 1996; Young et al., 2006), and emotional states (e.g., anger, anxiety, sadness) that are dominant and/or maladaptive in depressive disorders. The goal of the proposed study was to investigate differences in the speech content of depressed and healthy participants from different countries (Northern Ireland, Italy, and Russia) using a computerized text analysis tool, the Linguistic Inquiry Word Count (LIWC) tool (Pennebaker et al., 2001, 2003; Tausczik and Pennebaker, 2010) which classifies words of a given text into several categories. The novelty of our study, compared to previous studies, lies in:

- Cross Cultural Comparison: Its multilingual approach and cultural comparison across three different European areas. This aspect has not been sufficiently explored in the existing literature. Our main contribution lies in the analysis of cultural differences, which offers new perspectives on the manifestation of depression in different contexts.
- Innovative Discoveries: Our results point to an observation that could contribute to our understanding of depression from a linguistic standpoint. We observed that participants in the depressed group tended to speak more spontaneously and with less self-regulation compared to healthy subjects; to the best of our knowledge this effect has not been observed yet in literature.
- Use of Whisper: A new tool for automatic transcription of audio data. Its ability to surpass previous benchmarks for automatic multilingual speech recognition represents a significant technological advance.
- Clinical Implications: The data collected can inform the design of autonomous systems that can detect early signs of mood changes and depression through the analysis of interactional exchanges. This approach can facilitate automated, low-cost technological interventions for use in health centers and by mental health professionals.
- Relevance to Diagnosis and Treatment: Considering the importance of addressing misclassification for accurate diagnosis and treatment, recognizing and mitigating cultural biases can improve the validity of the research and ensure that assessments are comparable across diverse populations, leading to better mental health outcomes.

The Linguistic Inquiry and Word Count (LIWC-22) was chosen as the primary tool for automated language analysis because of its established reliability and validity in psychological research (Pennebaker et al., 2015). Unlike some other text analysis tools, which may focus on different aspects of language, LIWC-22 is specifically designed to analyze text on a word-by-word basis, categorizing words into psychologically relevant dimensions such as emotional tone, social orientation, and cognitive processes (Tausczik and Pennebaker, 2010). For example, tools like NLTK (Bird et al., 2009) in Python are powerful for tasks like part-of-speech tagging and syntactic parsing, while topic modeling techniques using LDA (Blei et al., 2003) identify latent topics within a text corpus. However, these tools do not provide the same level of insight into the psychological substructures of language as LIWC-22. This capability is particularly useful for our study, as it allows us to investigate subtle differences in language use that reflect the emotional and cognitive states associated with depression across different cultures. While other tools may offer broader linguistic analysis, LIWC-22 provides targeted insights into the specific psychological constructs relevant to our research questions.

## 2 Material and methods

### 2.1 Participants

The study involved a total of 241 participants, divided into three groups. Group 1 consisted of 65 native English speakers from Northern Ireland (UK), 30 healthy subjects with no history or current conditions of any psychiatric disorders (16 females and 14 males, mean age = 50.9; SD = ±11.7) and 35 participants diagnosed with Major depressive disorder (17 females and 18 males, mean age = 45.5; SD = ±13.9); the clinical group (diagnosed with depression) was recruited among Action Mental Health clients. Action Mental Health (AMH) is a Northern Irish non-profit organization which operates to improve the wellbeing of people with mental health needs and assisting them in their professional integration. Group 2 comprised 108 native Italian speakers, 54 healthy subjects with no history or current conditions of any psychiatric disorders (45 females and 9 males, mean age = 47.3; SD = ±11.7) and 54 participants diagnosed with Major depressive disorder (41 females and 13 males, mean age = 45.2; SD = ±12.4) recruited among the patients of six medical centers located within the Campania region (south of Italy). Group 3 consisted of 68 native Russian speakers, participants belonging to this group were school and college students (recruited from Moscow region and Komi republic, Russia) who had not been officially diagnosed with any kind of mental disorder, however after the administration of the DASS-21 (Lovibond and Lovibond, 1995; Ruzhenkova et al., 2019), a scale used to assess the current symptoms of Depression, Anxiety, and Stress, some participants' scores exceeded the cut-off values for depression, allowing us to divide the group in: 34 healthy subjects (18 females and 16 males, mean age = 17; SD = ±1.7) and 34 subjects with depressive symptoms (20 females and 14 males, mean age = 16.7; SD = ±1.4).

Table 1 provides a detailed overview of the participants involved in this study, divided into three main groups: Group 1 (Northern Ireland, UK), Group 2 (Italy), and Group 3 (Russia). Each group is further divided into healthy and depressed participants. The table columns include information on country of origin, number of healthy and depressed participants, participants' sex (females and males), mean age and standard deviation for each group.

**Table 1:** Participants' demographic and clinical characteristics.

**Category**	**Group 1**		**Group 2**		**Group 3**	
Country	Northern Ireland (UK)		Italy		Russia	
Participants	30 Healthy	35 Depressed	54 Healthy	54 Depressed	34 Healthy	34 Depressed
Sex	16 F;14 M	17 F; 18 M	45 F; 9 M	41 F; 13 M	18 F; 16 M	20 F; 14 M
Mean age	50.9	45.5	47.3	45.2	17	16.7
SD	±11.7	±13.9	±11.7	±12.4	±1.7	±1.4

### 2.2 Tools and procedures

To ensure quality data capture, participants were required to sit in front of a laptop equipped with a microphone and asked to complete the following tasks designed to accurately record the participants' voice features. Audio recordings via computer were collected in which the subjects were asked to read out loud a brief Aesop's fable, named “*The north wind and the sun*” (hereafter referred to as the “Tale Task”) from the laptop monitor and then to talk about how they spent the past week, or to narrate any event they considered relevant, and to speak for a minimum of 2 min (hereafter referred to as the “Diary Task”). Participants spoke in their native language when completing the verbal tasks. English (for Northern Ireland), Italian, and Russian.

### 2.3 Verbal behavior extraction

The content and the linguistic style of speech (Tausczik and Pennebaker, 2010) are two aspects that reflect cognitive patterns (e.g., self-focus, pessimism, low self-esteem; Beck et al., 1996; Young et al., 2006), and emotional states (e.g., anger, anxious, sadness) that are dominant and/or maladaptive in depressive disorders. For this reason, in order to identify and extract linguistic features which can serve to signal the presence of a depressive state, specific tools, described below, have been exploited on the diary task data, namely on audio recordings in which participants were free to talk about how they spent the past week or recounting any event they considered relevant. Audio data were transcribed using Whisper, an Automatic Speech Recognition (ASR) model produced by OpenAI (Radford et al., 2023). Whisper is the latest in a series of Convolutional Transformer End-to-End ASR models, similar in structure to Wav2Vec2 (Baevski et al., 2020) and WavLM (Chen et al., 2022). We use the pretrained Whisper Medium model for all of the automated transcriptions in this work. Whisper was trained on 680,000 h of multilingual audio data with transcription collected from the web. Ground truth transcriptions for the audio training data were collected from subtitle files accompanying the audio and filtered using various heuristics in an attempt to ensure ground truth quality, while striving for dataset scale. The resulting approach is termed weakly supervised, as each ground truth transcription is not human reviewed but is instead filtered automatically. The scale of the model and the effectiveness of the weak supervision have seen Whisper exceeding previous state of the art benchmarks for multilingual ASR, notable particularly as training sets of said benchmark corpora are not included in Whisper training data. Once transcribed, the text was analyzed using the LIWC-22 tool (Pennebaker et al., 2001, 2003; Tausczik and Pennebaker, 2010). Linguistic Inquiry and Word Count (LIWC) consists of a software for analyzing word use. It can be used to study a single individual, groups of people over time, or large social media datasets. LIWC-22 consists of software and a dictionary; within the text, starting from the dictionary's words, the software identifies and analyzes target words which allows the identification of language categories that fit into a particular domain (e.g., negative emotion words).

## 3 Data analysis and results

**Figure 1:**
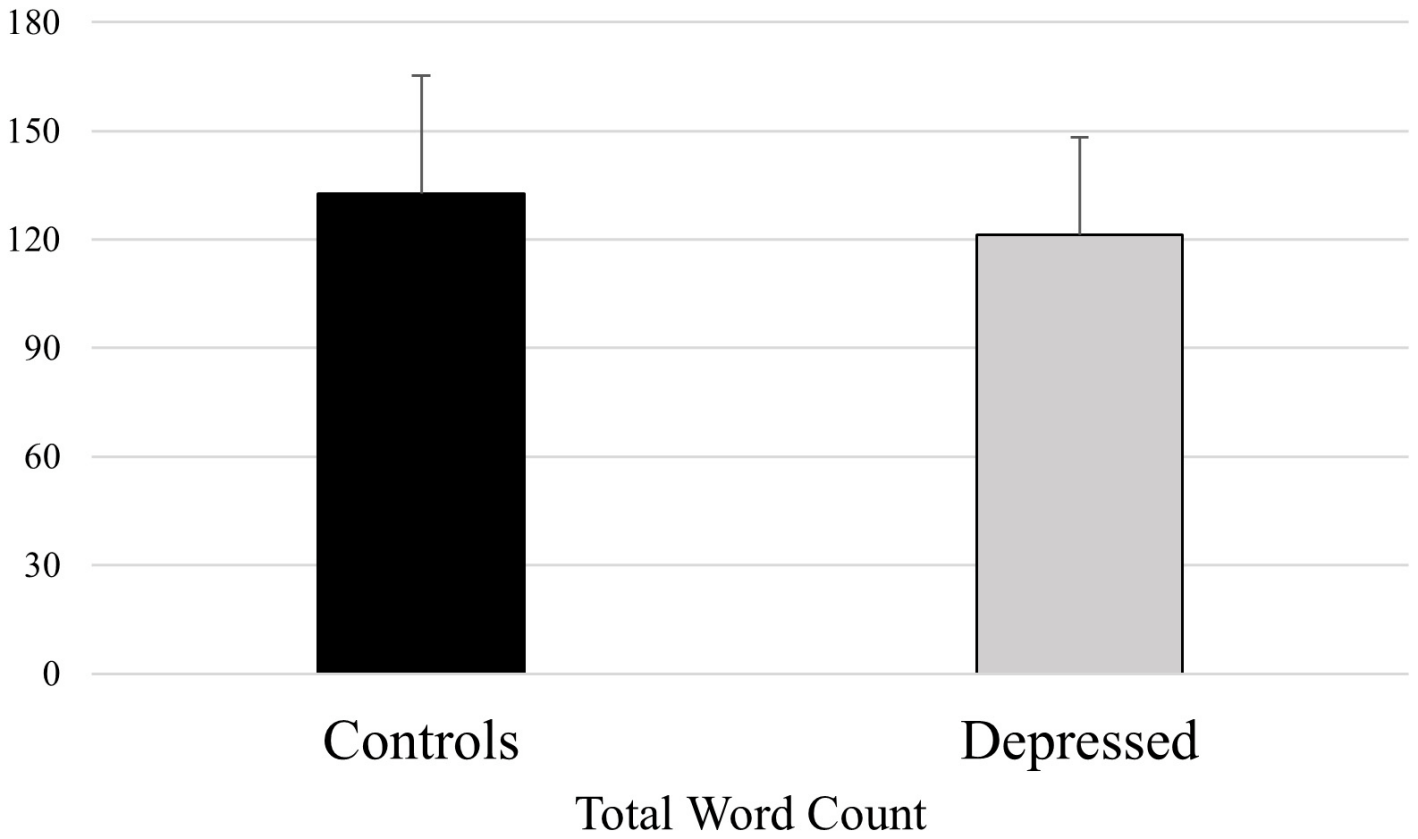
Mean differences between healthy controls and depressed participants for the LIWC variable Word Count (raw number of words used).

**Figure 2:**
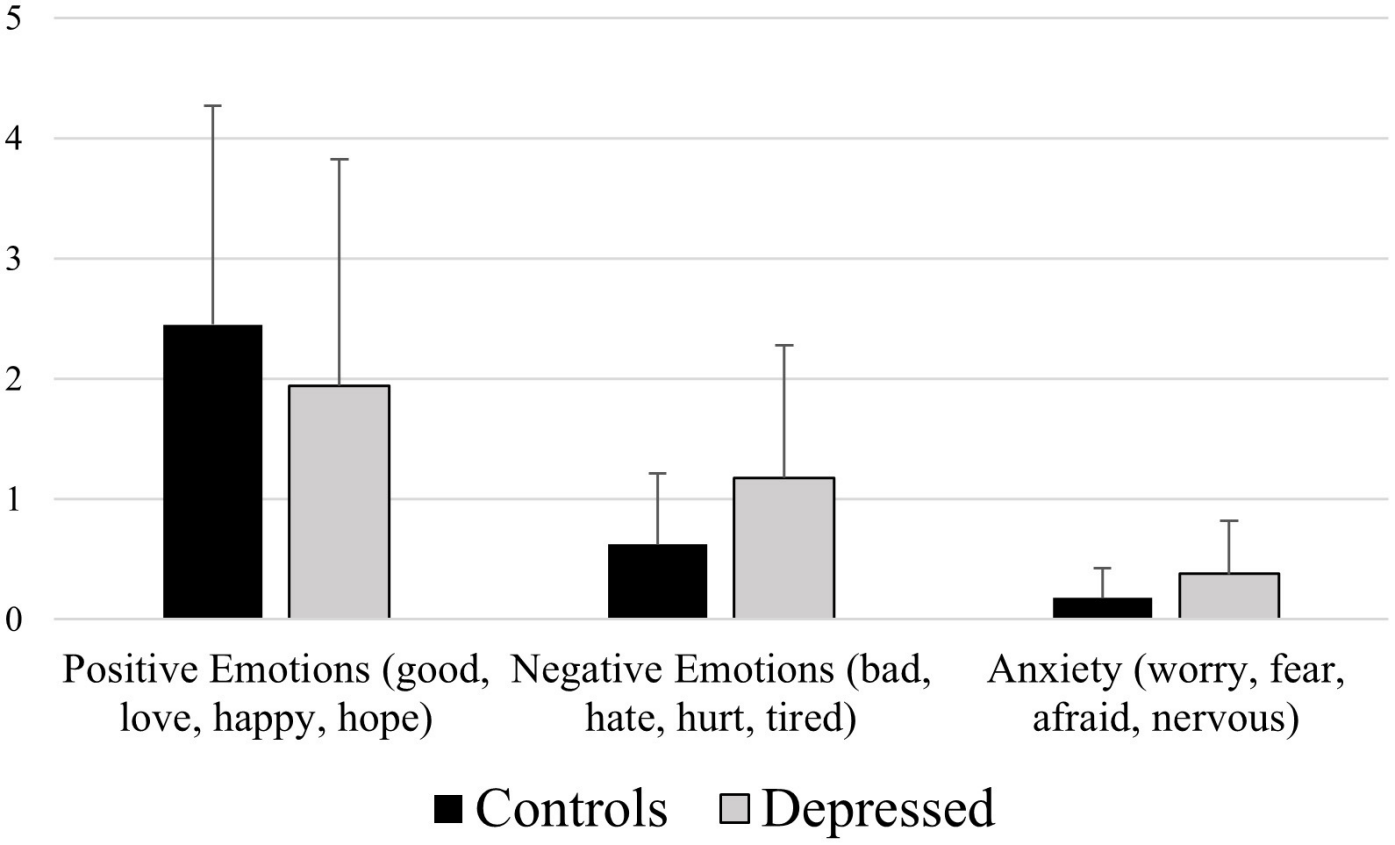
Mean differences between healthy controls and depressed participants for the LIWC variables Positive Emotions (e.g., “good,” “love,” “happy,” “hope”), Negative Emotions (e.g., “bad,” “hate,” “hurt,” “tired”), and Anxiety (e.g., “worry,” “fear,” “afraid,” “nervous”).

**Figure 3:**
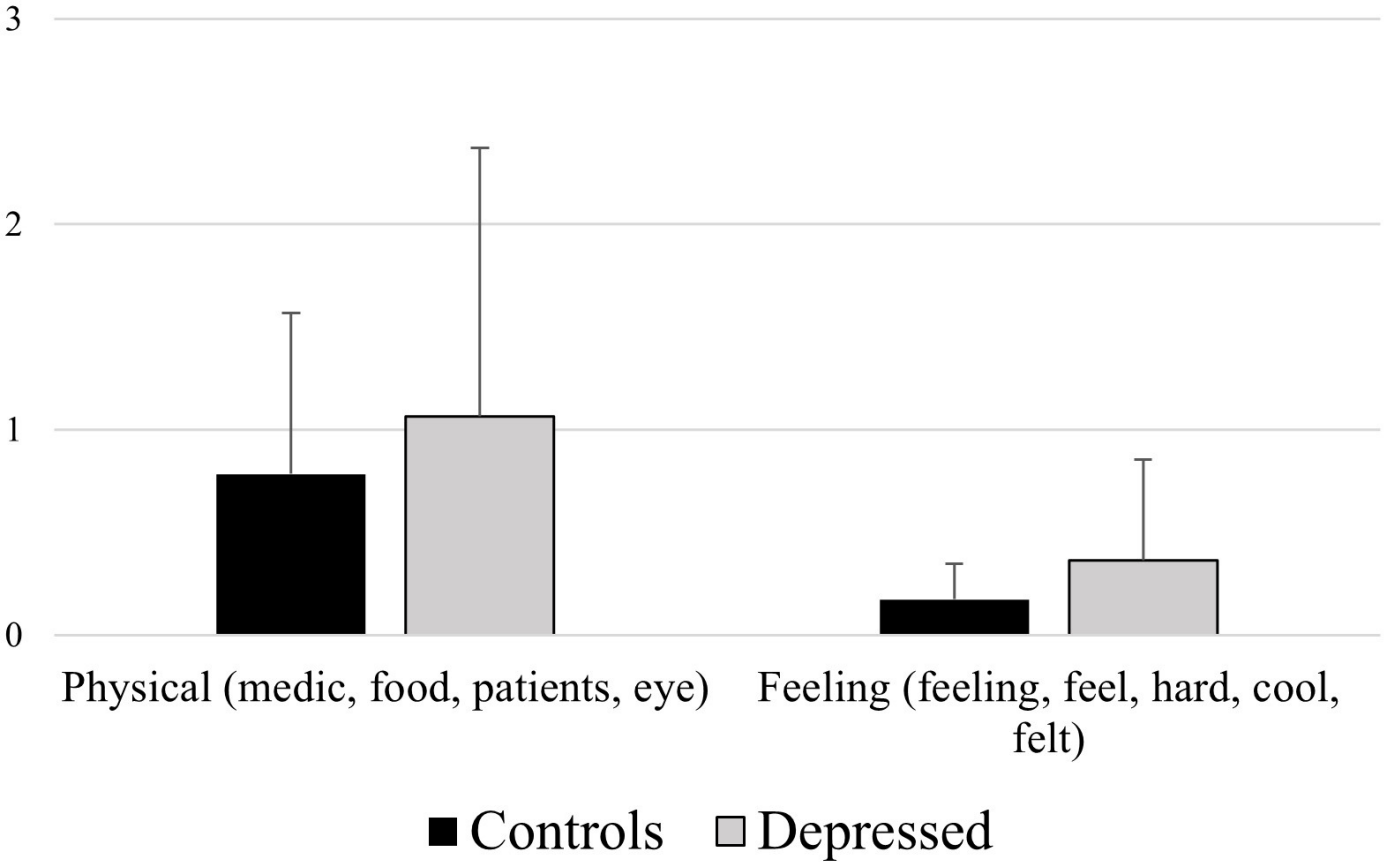
Mean differences between healthy controls and depressed participants for the LIWC variables Physical (e.g., “medic,” “food,” “patients,” “eye”) and Feeling (e.g., “feeling,” “feel,” “hard,” “cool,” “felt”).

**Figure 4:**
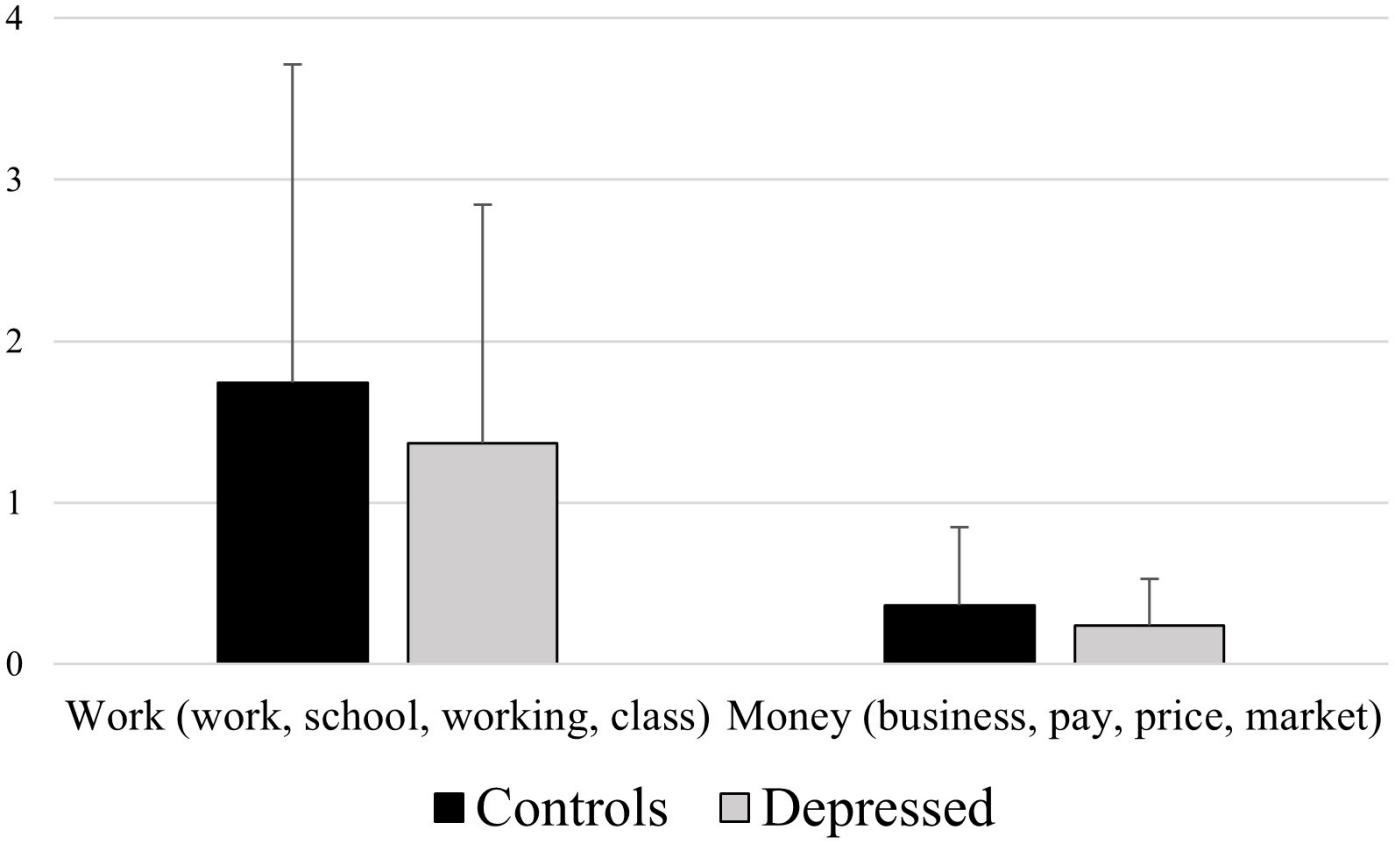
Mean differences between healthy controls and depressed participants for the LIWC variables Work (e.g., “work,” “school,” “working,” “class”) and Money (e.g., “business,” “pay,” “price,” “market”).

**Figure 5:**
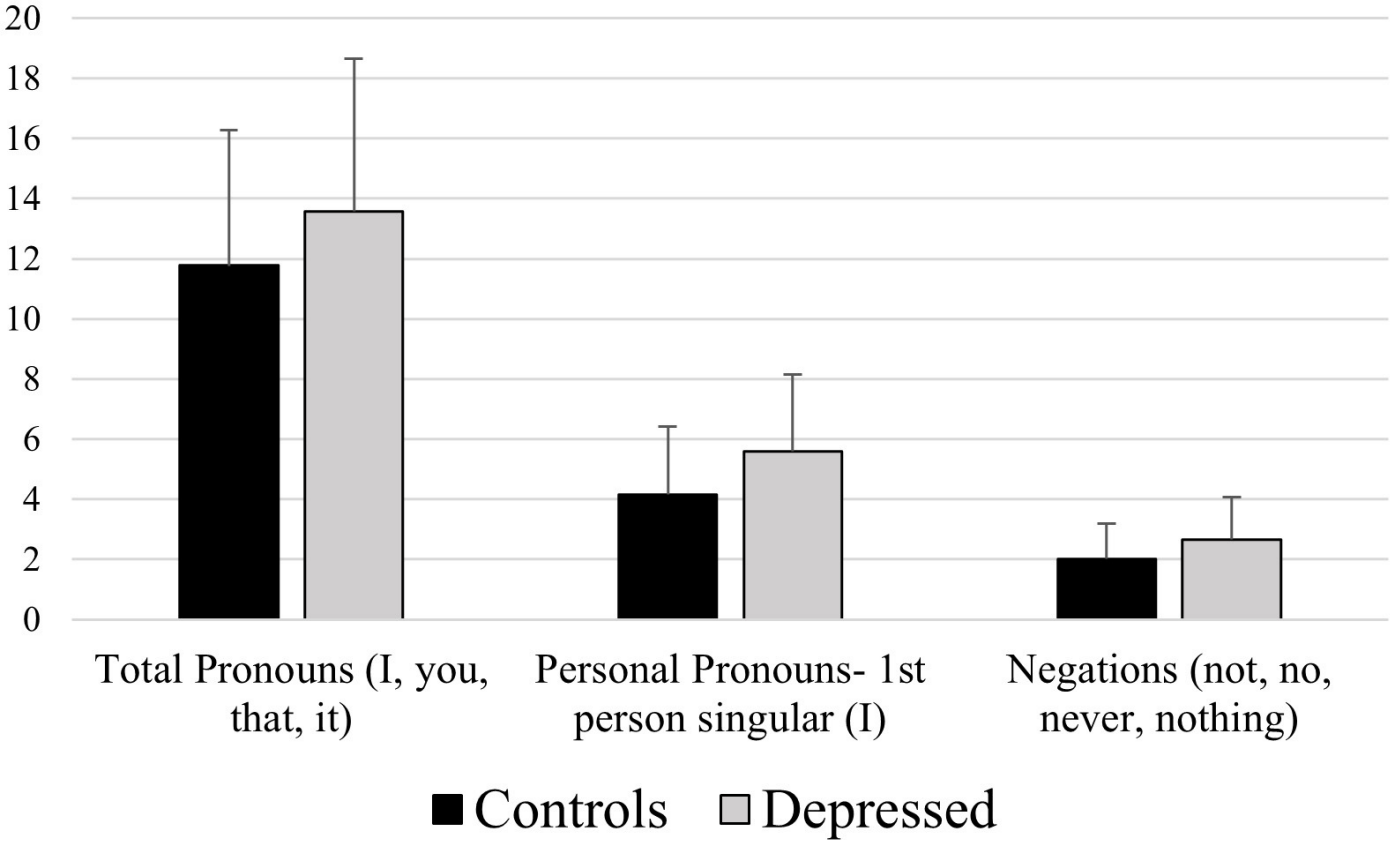
Mean differences between healthy controls and depressed participants for the LIWC variables Total Pronouns (e.g., “I,” “you,” “that,” “it”), Personal Pronouns−1st person singular (I), and Negations (e.g., “not,” “no,” “never,” “nothing”).

**Figure 6:**
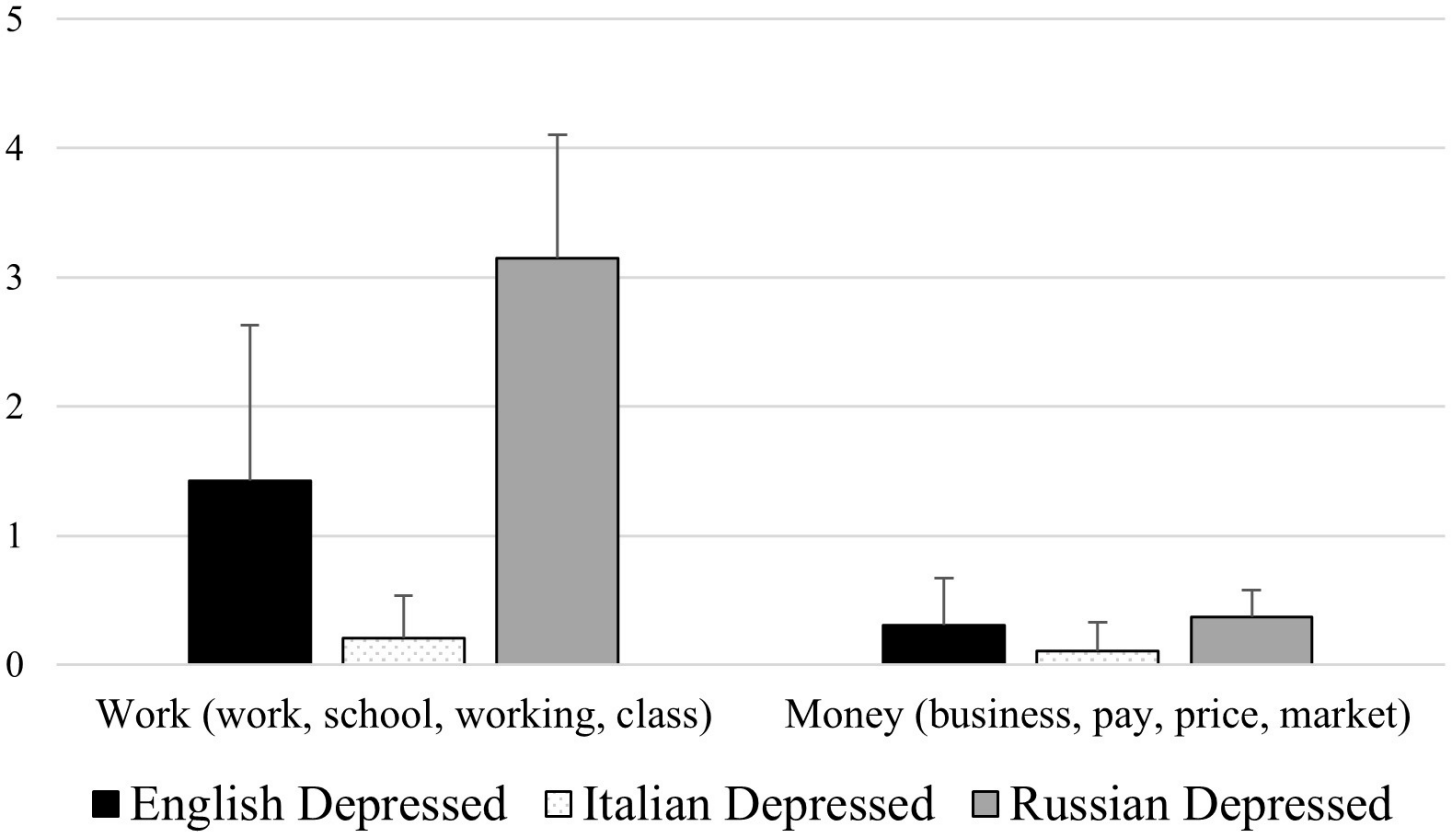
Mean differences among English, Italian, and Russian depressed participants for the LIWC variables Work (e.g., “work,” “school,” “working,” “class”) and Money (e.g., “business,” “pay,” “price,” “market”).

**Figure 7:**
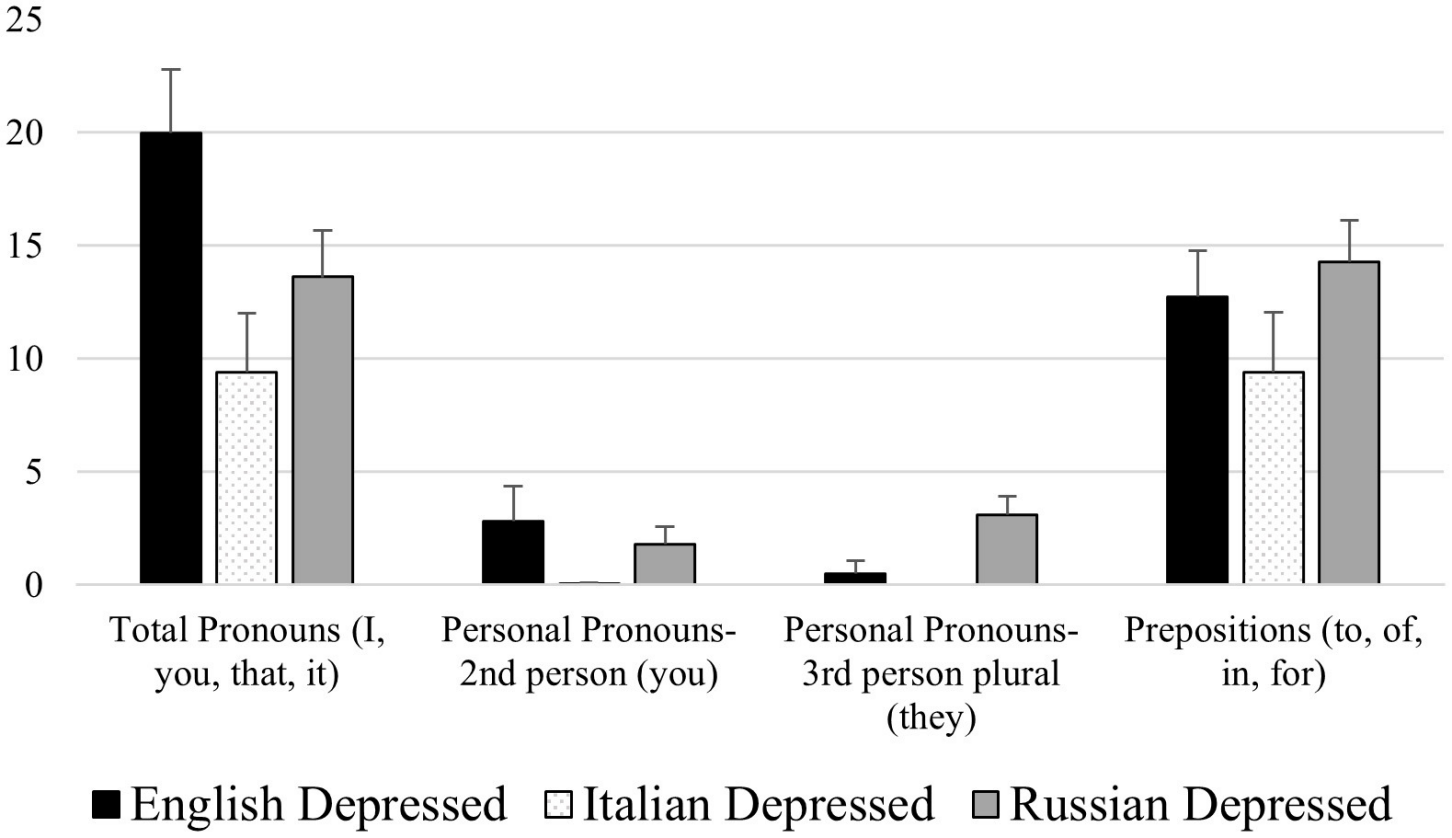
Mean differences among English, Italian, and Russian depressed participants for the LIWC variables Total Pronouns (“I,” “you,” “that,” “it”), 2nd and 3rd Person Personal Pronouns (“you” and “they”), and Prepositions (“to,” “of,” “in,” “for”).

The acquired data were analyzed using Multivariate Analysis of Variance (MANOVA). Participant's group (healthy controls and depressed participants) and country (English, Italian, and Russian) were studied as fixed factors. The mean of each LIWC-22 category was used as a dependent variable. The significance level α < 0.05 was adopted. Bonferroni's *post-hoc* tests were used to assess mean differences. MANOVA was chosen as statistical method for several reasons. First, our research questions required us to examine the effects of participant's group (healthy controls vs. depressed participants) and country (Northern Ireland, Italy, and Russia) on multiple linguistic features simultaneously, as measured by the LIWC-22 categories. MANOVA allowed us to analyze these dependent variables as a system, rather than individually, providing a more holistic understanding of the linguistic differences. Second, our study employed a factorial design with two between-subjects independent variables, and MANOVA is well-suited for analyzing main and interaction effects in such designs. Finally, MANOVA is appropriate when the dependent variables are correlated, as is often the case with different linguistic categories. Significant results for each LIWC-22 category are shown below, in Tables 2, 3. Following the tables a narrative description of the results is presented. Mean differences for some of the LIWC-22 categories are also graphically showed (see Figures 1–7).

**Table 2:** Significant results for each LIWC-22 category—group effects.

**LIWC-22 category**	**Fisher's *F***	**Controls' mean scores**	**Depressed mean scores**	**Alpha (α)**	**Eta-squared (η^2^)**
*Word count (raw number of words)*	*F*_(1, 129)_ = 17.173, *p* ≪ 0.01	135.368	119.892	*p* ≪ 0.01	0.070
*Big words (words with seven letters or longer)*	*F*_(1, 129)_ = 13.513, *p* ≪ 0.01	19.362	17.710	*p* ≪ 0.01	0.056
*Total pronouns (I, you, that, it)*	*F*_(1, 129)_ = 16.498, *p* ≪ 0.01	12.707	14.049	*p* ≪ 0.01	0.067
*Personal pronouns—first person singular (I)*	*F*_(1, 129)_ = 27.603, *p* ≪ 0.01	4.326	5.5994	*p* ≪ 0.01	0.108
*Negations (not, no, never, nothing)*	*F*_(1, 129)_ = 20.621, *p* ≪ 0.01	1.863	2.608	*p* ≪ 0.01	0.083
*Positive emotions (good, love, happy, hope)*	*F*_(1, 129)_ = 8.379, *p* = 0.004	2.573	2.185	*p* = 0.004	0.035
*Negative emotions (bad, hate, hurt, tired)*	*F*_(1, 129)_ = 13.709, *p* ≪ 0.01	0.593	0.990	*p* ≪ 0.01	0.056
*Anxiety (worry, fear, afraid, nervous)*	*F*_(1, 129)_ = 10.624, *p* = 0.001	0.189	0.346	*p* = 0.001	0.044
*Work (work, school, working, class)*	*F*_(1, 129)_ = 9.011, *p* = 0.003	2.084	1.638	*p* = 0.003	0.038
*Money (business, pay, price, market)*	*F*_(1, 129)_ = 11.966, *p* = 0.001	0.434	0.263	*p* = 0.001	0.050
*Physical (medic, food, patients, eye)*	*F*_(1, 129)_ = 6.075, *p* = 0.014	0.824	1.113	*p* = 0.014	0.026
*Visual (see, look, eye, saw)*	*F*_(1, 129)_ = 10.501, *p* = 0.001	0.548	0.335	*p* = 0.001	0.044
*Feeling (feeling, feel, hard, cool, felt)*	*F*_(1, 129)_ = 6.915, *p* = 0.009	0.175	0.316	*p* = 0.009	0.029

**Table 3:** Significant results for each LIWC-22 category—country effects.

**LIWC-22 category**	**Fisher's *F***	**English' mean scores**	**Italians' mean scores**	**Russian's mean scores**	**Alpha (α)**	**Eta-squared (η^2^)**
*Word count (raw number of words)*	*F*_(2, 129)_ = 27.060, *p* ≪ 0.01	143.607	129.574	109.709	*p* ≪ 0.01	0.191
*Words per sentence (average words per sentence)*	*F*_(2, 129)_ = 4.460, *p* = 0.013	12.885	41.675	69.194	*p* = 0.013	0.037
*Big words (words with seven letters or longer)*	*F*_(2, 129)_ = 277.559, *p* ≪ 0.01	11.612	19.293	24.704	*p* ≪ 0.01	0.708
*Total pronouns (I, you, that, it)*	*F*_(2, 129)_ = 322.374, *p* ≪ 0.01	18.703	8.465	12.967	*p* ≪ 0.01	0.738
*Personal pronouns—first person singular (I)*	*F*_(2, 129)_ = 108.466, *p* ≪ 0.01	7.510	3.848	3.529	*p* ≪ 0.01	0.486
*Personal pronouns-first person plural (we)*	*F*_(2, 129)_ = 10.937, *p* ≪ 0.01	0.973	0.484	0.538	*p* ≪ 0.01	0.087
*Personal pronouns—second person (you)*	*F*_(2, 129)_ = 144.621, *p* ≪ 0.01	2.273	0.040	2.069	*p* ≪ 0.01	0.558
*Personal pronouns−3rd person singular (he/she)*	*F*_(2, 129)_ = 32.099, *p* ≪ 0.01	1.280	0.175	1.416	*p* ≪ 0.01	0.219
*Personal pronouns−3rd person plural (they)*	*F*_(2, 129)_ = 319.477, *p* ≪ 0.01	0.729	0	2.923	*p* ≪ 0.01	0.736
*Numbers (one, two, first, once)*	*F*_(2, 129)_ = 61.048, *p* ≪ 0.01	1.504	0.298	0.757	*p* ≪ 0.01	0.348
*Prepositions (to, of, in, for)*	*F*_(2, 129)_ = 117.339, *p* ≪ 0.01	13.233	9.158	14.286	*p* ≪ 0.01	0.506
*Negations (not, no, never, nothing)*	*F*_(2, 129)_ = 25.319, *p* ≪ 0.01	1.405	2.564	2.737	*p* ≪ 0.01	0.181
*Positive emotions (good, love, happy, hope)*	*F*_(2, 129)_ = 312.553, *p* ≪ 0.01	1.161	1.220	4.757	*p* ≪ 0.01	0.732
*Negative emotions (bad, hate, hurt, tired)*	*F*_(2, 129)_ = 27.562, *p* ≪ 0.01	0.390	1.326	0.658	*p* ≪ 0.01	0.194
*Anxiety (worry, fear, afraid, nervous)*	*F*_(2, 129)_ = 9.810, *p* ≪ 0.01	0.153	0.236	0.413	*p* ≪ 0.01	0.079
*Anger (hate, mad, angry)*	*F*_(2, 129)_ = 16.775, *p* ≪ 0.01	0.006	0.279	0.197	*p* ≪ 0.01	0.128
*Sadness (sad, disappointed, cry)*	*F*_(2, 129)_ = 15.069, *p* ≪ 0.01	0.059	0.461	0.111	*p* ≪ 0.01	0.116
*Friends (friend, boyfriend, girlfriend, dude)*	*F*_(2, 129)_ = 33.429, *p* ≪ 0.01	0.097	0.203	0.536	*p* ≪ 0.01	0.226
*Home (home, house, room, bed)*	*F*_(2, 129)_ = 14.634, *p* ≪ 0.01	0.819	1.194	0.469	*p* ≪ 0.01	0.113
*Work (work, school, working, class)*	*F*_(2, 129)_ = 160.411, *p* ≪ 0.01	1.927	0.221	3.436	*p* ≪ 0.01	0.584
*Money (business, pay, price, market)*	*F*_(2, 129)_ = 20.120, *p* ≪ 0.01	0.483	0.133	0.430	*p* ≪ 0.01	0.149
*Physical (medic, food, patients, eye)*	*F*_(2, 129)_ = 78.834, *p* ≪ 0.01	1.933	0.854	0.119	*p* ≪ 0.01	0.408
*Sexual (sex, gay, pregnant)*	*F*_(2, 129)_ = 7.892 *p* ≪ 0.01	0	0.225	0.154	*p* ≪ 0.01	0.064
*Motion (go, come, went, came)*	*F*_(2, 129)_ = 321.521 *p* ≪ 0.01	2.199	1.830	6.139	*p* ≪ 0.01	0.737
*Space (in, out, up, there)*	*F*_(2, 129)_ = 435.203, *p* ≪ 0.01	8.212	0.838	9.427	*p* ≪ 0.01	0.792
*Visual (see, look, eye, saw)*	*F*_(2, 129)_ = 8.945, *p* ≪ 0.01	0.641	0.343	0.342	*p* ≪ 0.01	0.072
*Auditory (sound, heard, hear, music)*	*F*_(2, 129)_ = 35.663, *p* ≪ 0.01	0.157	0.895	0.854	*p* ≪ 0.01	0.237
*Time (when, now, then, day)*	*F*_(2, 129)_ = 47.547, *p* ≪ 0.01	6.844	4.204	7.495	*p* ≪ 0.01	0.293

Table 2 summarizes significant results obtained from the linguistic analysis concerning the effects of participant's group (healthy vs. depressed). The table is organized into five main columns: the first column lists the language categories analyzed using LIWC-22, the second column shows the Fisher's *F* statistic, the third column shows the average mean scores for the control group, the fourth column shows the average mean scores for the depressed group, the fifth column shows the significance level alpha (α), and the sixth column shows Eta-squared (η^2^) values measuring the effect size. Only LIWC-22 categories where significant effects were observed are reported.

Table 3 summarizes significant results obtained from the linguistic analysis concerning the effects of the country (Northern Ireland, Italy, and Russia). The table is organized into seven main columns: the first column lists the language categories analyzed using LIWC-22, the second column shows the Fisher's *F* statistic, the third column shows the average mean scores for the English group, the fourth column shows the average mean scores for the Italian group, the fifth column shows the average mean scores for the Russian group, the sixth column shows the significance level alpha (α), and the seventh column shows Eta-squared (η^2^) values measuring the effect size. Only LIWC-22 categories where significant effects were observed are reported. *Word Count (raw number of words)*

A significant effect of participants' group emerged [*F*_(1, 129)_ = 17.173, *p* ≪ 0.01], revealing controls' higher means (135.368) than depressed participants (119.892), *p* ≪ 0.01.

A significant effect of participants' country emerged [*F*_(2, 129)_ = 27.060, *p* ≪ 0.01], with English participants showing higher means (143.607), than Italians (129.574, *p* = 0.007) and Russian (109.709, *p* ≪ 0.01).

A significant interaction emerged between participants' group and country [*F*_(2, 129)_ = 3.197, *p* = 0.043]. Bonferroni *post-hoc* tests were singularly performed on each variable and highlighted that:

a) Concerning participants' groups: English healthy controls showed higher means (156.626) compared to English depressed participants (130.589, *p* ≪ 0.01), and Italian healthy controls showed higher means (138.319) compared to Italian depressed participants (120.830, *p* = 0.006).
b) Concerning participants' country: Russian healthy controls showed lower means (111.159) compared to English (156.626, *p* ≪ 0.01) and Italians (138.319, *p* ≪ 0.01) controls. Russian depressed participants showed significantly lower means (108.258) compared to English depressed participants (130.589, *p* = 0.002).

*Words per sentence (average words per sentence)*

A significant effect of participants' country emerged [*F*_(2, 129)_ = 4.460, *p* = 0.013], with Russian participants showing higher means (69.194), than English (12.885, *p* = 0.009).

*Big Words (words with seven letters or longer)*

A significant effect of participants' group emerged [*F*_(1, 129)_ = 13.513, *p* ≪ 0.01], showing controls' higher means (19.362) than depressed participants (17.710), *p* ≪ 0.01.

A significant effect of participants' country emerged [*F*_(2, 129)_ = 277.559, *p* ≪ 0.01], showing Russian participants' higher means (24.704), than Italians (19.293, *p* ≪ 0.01) and English (11.612, *p* ≪ 0.01).

A significant interaction emerged between participants' group and country [*F*_(2, 129)_ = 3.862, *p* = 0.022]. Bonferroni *post-hoc* tests were singularly performed on each variable and highlighted that:

a) Concerning participants' groups: English healthy controls showed higher means (13.189) compared to English depressed participants (10.034, *p* ≪ 0.01), and Italian healthy controls showed higher means (20.163) compared to Italian depressed participants (18.423, *p* = 0.022).
b) Concerning participants' country: Russian healthy controls showed higher means (24.734) compared to English (13.189, *p* ≪ 0.01) and Italians (20.163, *p* ≪ 0.01) controls. Russian depressed participants showed significantly higher means (24.674) compared to English (10.034, *p* ≪ 0.01) and Italian (18.423, *p* ≪ 0.01) depressed participants.

*Total Pronouns (I, you, that, it)*

A significant effect of participants' group emerged [*F*_(1, 129)_ = 16.498, *p* ≪ 0.01], revealing depressed participants' higher means (14.049) than controls (12.707), *p* ≪ 0.01.

A significant effect of participants' country emerged [*F*_(2, 129)_ = 322.374, *p* ≪ 0.01], showing English participants' higher means (18.703), than Italians (8.465, *p* ≪ 0.01) and Russian (12.967, *p* ≪ 0.01).

A significant interaction emerged between participants' group and country [*F*_(2, 129)_ = 3.142, *p* = 0.043]. Bonferroni *post-hoc* tests were singularly performed on each variable and highlighted that:

a) Concerning participants' groups: English healthy controls showed lower means (17.449) compared to English depressed participants (19.956, *p* ≪ 0.01).
b) Concerning participants' country: English healthy controls showed higher means (17.449) compared to Italians (8.165, *p* ≪ 0.01) and Russian (12.508, *p* ≪ 0.01) controls. English depressed participants showed significantly higher means (19.956) compared to Italian (8.765, *p* ≪ 0.01) and Russian (13.425, *p* ≪ 0.01) depressed participants.

*Personal Pronouns—first person singular (I)*

A significant effect of participants' group emerged [*F*_(1, 129)_ = 27.603, *p* ≪ 0.01], revealing depressed participants' higher means (5.5994) than controls (4.326), *p* ≪ 0.01.

A significant effect of participants' country emerged [*F*_(2, 129)_ = 108.466, *p* ≪ 0.01], showing English participants' higher means (7.510), than Italians (3.848, *p* ≪ 0.01) and Russian (3.529, *p* ≪ 0.01).

*Personal Pronouns-first person plural (we)*

A significant effect of participants' country emerged [*F*_(2, 129)_ = 10.937, *p* ≪ 0.01], showing English participants' higher means (0.973), than Italians (0.484, *p* ≪ 0.01) and Russian (0.538, *p* = 0.001).

*Personal Pronouns—second person (you)*

A significant effect of participants' country emerged [*F*_(2, 129)_ = 144.621, *p* ≪ 0.01], showing Italian participants' lower means (0.040), than English (2.273, *p* ≪ 0.01) and Russian (2.069, *p* ≪ 0.01).

A significant interaction emerged between participants' group and country [*F*_(2, 129)_ = 15.210, *p* ≪ 0.01]. Bonferroni *post-hoc* tests were singularly performed on each variable and highlighted that:

a) Concerning participants' groups: English healthy controls showed lower means (1.714) compared to English depressed participants (2.833, *p* ≪ 0.01) and Russian healthy controls showed higher means (2.306) compared to Russian depressed participants (1.833, *p* = 0.025)
b) Concerning participants' country: Italian healthy controls showed lower means (0.059) compared to English (1.714, *p* ≪ 0.01) and Russian (2.306, *p* ≪ 0.01) controls. Italian depressed participants showed significantly lower means (0.021) compared to English (2.833, *p* ≪ 0.01) and Russian (1.833, *p* ≪ 0.01) depressed participants.

*Personal Pronouns*−*3rd person singular (he/she)*

A significant effect of participants' country emerged [*F*_(2, 129)_ = 32.099, *p* ≪ 0.01], showing Italian participants' lower means (0.175), than English (1.280, *p* ≪ 0.01) and Russian (1.416, *p* ≪ 0.01).

*Personal Pronouns*−*3rd person plural (they)*

A significant effect of participants' country emerged [*F*_(2, 129)_ = 319.477, *p* ≪ 0.01], showing Italian participants' lower means (0), than English (0.729, *p* ≪ 0.01) and Russian (2.923, *p* ≪ 0.01).

A significant interaction emerged between participants' group and country [*F*_(2, 129)_ = 4.384, *p* = 0.014]. Bonferroni *post-hoc* tests were singularly performed on each variable and highlighted that:

a) Concerning participants' groups: English healthy controls showed higher means (0.973) compared to English depressed participants (0.484, *p* = 0.006).
b) Concerning participants' country: Italian healthy controls showed lower means (0) compared to English (0.973, *p* ≪ 0.01) and Russian (2.812, *p* ≪ 0.01) controls. Italian depressed participants showed significantly lower means (0) compared to English (0.484, *p* ≪ 0.01) and Russian (3.033, *p* ≪ 0.01) depressed participants.

*Numbers (one, two, first, once)*

A significant effect of participants' country emerged [*F*_(2, 129)_ = 61.048, *p* ≪ 0.01], showing Italian participants' lower means (0.298), than English (1.504, *p* ≪ 0.01) and Russian (0.757, *p* ≪ 0.01).

*Prepositions (to, of, in, for)*

A significant effect of participants' country emerged [*F*_(2, 129)_ = 117.339, *p* ≪ 0.01], showing Italian participants' lower means (9.158), than English (13.233, *p* ≪ 0.01) and Russian (14.286 *p* ≪ 0.01).

A significant interaction emerged between participants' group and country [*F*_(2, 129)_ = 5.494, *p* = 0.005]. Bonferroni *post-hoc* tests were singularly performed on each variable and highlighted that:

a) Concerning participants' groups: Italian healthy controls showed lower means (8.482) compared to Italian depressed participants (9.834, *p* = 0.007).
b) Concerning participants' country: Italian healthy controls showed lower means (8.482) compared to English (13.731, *p* ≪ 0.01) and Russian (14.324, *p* ≪ 0.01) controls. Italian depressed participants showed significantly lower means (9.834) compared to English (12.736, *p* ≪ 0.01) and Russian (14.247, *p* ≪ 0.01) depressed participants.

*Negations (not, no, never, nothing)*

A significant effect of participants' group emerged [*F*_(1, 129)_ = 20.621, *p* ≪ 0.01], revealing depressed participants' patients' higher means (2.608) than controls (1.863), *p* ≪ 0.01.

A significant effect of participants' country emerged [*F*_(2, 129)_ = 25.319, *p* ≪ 0.01], showing English participants' lower means (1.405), than Italians (2.564, *p* ≪ 0.01) and Russian (2.737 *p* ≪ 0.01).

A significant interaction emerged between participants' group and country [*F*_(2, 129)_ = 4.451, *p* = 0.013]. Bonferroni *post-hoc* tests were singularly performed on each variable and highlighted that:

a) Concerning participants' groups: Italian healthy controls showed lower means (1.929) compared to Italian depressed participants (3.200, *p* ≪ 0.01). English healthy controls showed lower means (0.973) compared to English depressed participants (1.838, *p* = 0.003).
b) Concerning participants' country: English healthy controls showed lower means (0.973) compared to Italian (1.929, *p* = 0.004) and Russian (2.688, *p* ≪ 0.01) controls. English depressed participants showed significantly lower means (1.838) compared to Italian (3.200, *p* ≪ 0.01) and Russian (2.787, *p* = 0.003) depressed participants.

*Positive Emotions (good, love, happy, hope)*

A significant effect of participants' group emerged [*F*_(1, 129)_ = 8.370, *p* = 0.004], revealing control participants' higher means (2.573) than depressed ones (2.185), *p* = 0.004.

A significant effect of participants' country emerged [*F*_(2, 129)_ = 312.553, *p* ≪ 0.01], showing English participants' lower means (1.161), than Italians (1.220, *p* ≪ 0.01) and Russian (4.757 *p* ≪ 0.01).

*Negative Emotions (bad, hate, hurt, tired)*

A significant effect of participants' group emerged [*F*_(1, 129)_ = 13.709, *p* ≪ 0.01], revealing depressed participants' higher means (0.990) than controls (0.593), *p* ≪ 0.01.

A significant effect of participants' country emerged [*F*_(2, 129)_ = 27.562, *p* ≪ 0.01], showing English participants' lower means (0.390), than Italians (1.326, *p* ≪ 0.01) and Russian (0.658 *p* ≪ 0.01).

*Anxiety (worry, fear, afraid, nervous)*

A significant effect of participants' group emerged [*F*_(1, 129)_ = 10.624, *p* = 0.001], revealing depressed participants' higher means (0.346) than controls (0.189), *p* = 0.001.

A significant effect of participants' country emerged [*F*_(2, 129)_ = 9.810, *p* ≪ 0.01], showing Russian participants' higher means (0.413), than Italians (0.236, *p* = 0.009) and English (0.153, *p* ≪ 0.01).

*Anger (hate, mad, angry)*

A significant effect of participants' country emerged [*F*_(2, 129)_ = 16.775, *p* ≪ 0.01], showing English participants' lower means (0.006), than Italians (0.279, *p* ≪ 0.01) and Russian (0.197, *p* ≪ 0.01).

*Sadness (sad, disappointed, cry)*

A significant effect of participants' country emerged [*F*_(2, 129)_ = 15.069, *p* ≪ 0.01], showing English participants' lower means (0.059), than Italians (0.461, *p* ≪ 0.01) and Russian (0.111, *p* ≪ 0.01).

*Friends (friend, boyfriend, girlfriend, dude)*

A significant effect of participants' country emerged [*F*_(2, 129)_ = 33.429, *p* ≪ 0.01], showing English participants' lower means (0.097), than Italians (0.203, *p* ≪ 0.01) and Russian (0.536, *p* ≪ 0.01).

*Home (home, house, room, bed)*

A significant effect of participants' country emerged [*F*_(2, 129)_ = 14.634, *p* ≪ 0.01], showing Russian participants' lower means (0.469), than Italians (1.194, *p* ≪ 0.01) and English (0.819 *p* = 0.035).

*Work (work, school, working, class)*

A significant effect of participants' group emerged [*F*_(1, 129)_ = 9.011, *p* = 0.003], revealing control participants' higher means (2.084) than depressed ones (1.638), *p* = 0.003

A significant effect of participants' country emerged [*F*_(2, 129)_ = 160.411, *p* ≪ 0.01], showing Italian participants' lower means (0.221), than English (1.927, *p* ≪ 0.01) and Russian (3.436, *p* ≪ 0.01).

A significant interaction emerged between participants' group and country [*F*_(2, 129)_ = 4.137, *p* = 0.017]. Bonferroni *post-hoc* tests were singularly performed on each variable and highlighted that:

a) Concerning participants' groups: English healthy controls showed higher means (2.426) compared to English depressed participants (1.427, *p* ≪ 0.01).
b) Concerning participants' country: Italian healthy controls showed lower means (0.201) compared to English (2.426, *p* ≪ 0.01) and Russian (3.625, *p* ≪ 0.01) controls. Italian depressed participants showed significantly lower means (0.242) compared to English (1.427, *p* ≪ 0.01) and Russian (3.246, *p* ≪ 0.01) depressed participants.

*Money (business, pay, price, market)*

A significant effect of participants' group emerged [*F*_(1, 129)_ = 11.966, *p* = 0.001], revealing control participants' higher means (0.434) than depressed ones (0.263), *p* = 0.001.

A significant effect of participants' country emerged [*F*_(2, 129)_ = 20.120, *p* ≪ 0.01], showing Italian participants' lower means (0.133), than English (0.483, *p* ≪ 0.01) and Russian (0.430, *p* ≪ 0.01).

A significant interaction emerged between participants' group and country [*F*_(2, 129)_ = 3.310, *p* = 0.038]. Bonferroni *post-hoc* tests were singularly performed on each variable and highlighted that:

a) Concerning participants' groups: English healthy controls showed higher means (0.658) compared to English depressed participants (0.308, *p* ≪ 0.01).
b) Concerning participants' country: Italian healthy controls showed lower means (0.164) compared to English (0.658, *p* ≪ 0.01) and Russian (0.479, *p* = 0.001) controls. Italian depressed participants showed significantly lower means (0.101) compared to English (0.308, *p* = 0.034) and Russian (0.381, *p* = 0.002) depressed participants.

*Physical (medic, food, patients, eye)*

A significant effect of participants' group emerged [*F*_(1, 129)_ = 6.075, *p* = 0.014], revealing depressed participants' higher means (1.113) than controls (0.824), *p* = 0.014.

A significant effect of participants' country emerged [*F*_(2, 129)_ = 78.834, *p* ≪ 0.01], showing Russian participants' lower means (0.119), than Italians (0.854, *p* ≪ 0.01) and English (1.933, *p* ≪ 0.01).

*Sexual (sex, gay, pregnant)*

A significant effect of participants' country emerged [*F*_(2, 129)_ = 7.892 *p* ≪ 0.01], showing English participants' lower means (0), than Italians (0.225, *p* ≪ 0.01) and Russian (0.154, *p* = 0.027).

*Motion (go, come, went, came)*

A significant effect of participants' country emerged [*F*_(2, 129)_ = 321.521 *p* ≪ 0.01], showing Italian participants' lower means (1.830), than English (2.199, *p* ≪ 0.01) and Russian (6.139, *p* ≪ 0.01).

*Space (in, out, up, there)*

A significant effect of participants' country emerged [*F*_(2, 129)_ = 435.203, *p* ≪ 0.01], showing Italian participants' lower means (0.838), than English (8.212, *p* ≪ 0.01) and Russian (9.427, *p* ≪ 0.01).

*Visual (see, look, eye, saw)*

A significant effect of participants' group emerged [*F*_(1, 129)_ = 10.501, *p* = 0.001], revealing control participants' higher means (0.548) than patients (0.335), *p* = 0.001.

A significant effect of participants' country emerged [*F*_(2, 129)_ = 8.945, *p* ≪ 0.01], showing English participants' higher means (0.641), than Italians (0.343, *p* = 0.001) and Russian (0.342, *p* = 0.001).

A significant interaction emerged between participants' group and country [*F*_(2, 129)_ = 5.010, *p* = 0.007]. Bonferroni *post-hoc* tests were singularly performed on each variable and highlighted that:

a) Concerning participants' groups: English healthy controls showed higher means (0.886) compared to English depressed participants (0.395, *p* ≪ 0.01).
b) Concerning participants' country: Russian healthy controls showed lower means (0.331) compared to English (0.886, *p* ≪ 0.01) and Italian (0.428, *p* ≪ 0.01) controls.

*Auditory (sound, heard, hear, music)*

A significant effect of participants' country emerged [*F*_(2, 129)_ = 35.663, *p* ≪ 0.01], showing English participants' lower means (0.157), than Italians (0.895, *p* ≪ 0.01) and Russian (0.854 *p* ≪ 0.01).

*Feeling (feeling, feel, hard, cool, felt)*

A significant effect of participants' group emerged [*F*_(1, 129)_ = 6.915, *p* = 0.001], revealing depressed participants' higher means (0.316) than controls (0.175),*p* = 0.009.

*Time (when, now, then, day)*

A significant effect of participants' country emerged [*F*_(2, 129)_ = 47.547, *p* ≪ 0.01], showing Italian participants' lower means (4.204), than English (6.844, *p* ≪ 0.01) and Russian (7.495, *p* ≪ 0.01).

## 4 Discussion

The study investigated differences in the speech content of depressed subjects and healthy participants from different countries (Northern Ireland, Italy, and Russia), exploiting verbal behavior analysis, which allowed the investigation of both the content and the linguistic style, using the computerized text analysis tool “LIWC” (Linguistic Inquiry Word Count). The aim of the study consisted in both evaluating speech content differences between depressed and healthy participants, as well as testing the effect of the culture on the linguistic manifestation of depression.

We observed that depressed patients, compared to healthy controls, showed a lower total Word Count, which indicates the raw number of used words, and a reduced usage of Big Words (words with seven letters or longer), confirming the tendency of depressed people in showing a decreased verbal production (see Figure 1), as already highlighted by other studies (Bagby et al., 2000). Our study also highlighted that depressed subjects, compared to controls, tended to use a more negative tone while speaking (greater use of words such as “bad,” “hate,” “hurt,” “tired”) and words related to negative emotions and anxiety (“worry,” “fear,” “afraid,” “nervous”), while controls expressed a more positive tone (higher use of words such as “good,” “love,” “happy,” “hope”; see Figure 2). Depressed people's tendency to exhibit an orientation toward a language characterized by negative emotions also emerged in other studies. Ramirez-Esparza et al. (2008) analyzed word use in depression forums and compared them with other typologies of forums, observing that women from depressed forums used more negative emotion words. A similar language pattern characterized by negative emotions was also observed within self-descriptions of people with subclinical depression (Newman et al., 2003) and analyzing Twitter's messages of users with major depressive symptoms (Rodriguez et al., 2010). This linguistic style is justified by cognitive mechanisms in which depressed individuals exhibit increased negative thinking and tend to focus more on negative aspects of their life (Beck, 1979; Park et al., 2012).

Our results also highlighted that depressed participants showed higher usage of first-person singular pronoun (I) compared to healthy controls (see Figure 3). This phenomenon has been widely observed in literature (Pyszczynski and Greenberg, 1987; Fernandez-Cabana et al., 2013; Wang et al., 2013; Bernard et al., 2016) and can be explained referring to the social engagement and disengagement model of depression theorized by Durkheim (1951), according to which depressed subjects tend to focus more on themselves and detach from others, in other words depression seem to be linked to an increased self-focused attention (Brockmeyer et al., 2015; Holtzman, 2017). However, depressed participants' higher usage of first-person singular pronoun (I) may also reflect a tendency to use self-references in their speech, which has been associated with authenticity (Markowitz et al., 2023). This could suggest that depressed subjects may exhibit a tendency to reveal themselves more directly, speaking more spontaneously and with less self-regulation compared to healthy subjects. This interpretation, however, should be considered exploratory and requires further investigation.

Results also highlighted depressed participants' higher general usage of pronouns (“I,” “you,” “that,” “it”) and negations (“not,” “no,” “never,” “nothing”; see Figure 3); this language style reflects depressed participants' tendency to adopt a particular type of thinking defined as “informal”. Informal type of thinking, focused on people and actions, is opposite to analytic thinking, characterized by formal, logical, and hierarchical thinking patterns (Jordan et al., 2019). A similar result was observed in a study (Smirnova et al., 2018) in which from the analysis of written reports from clinical group and healthy controls emerged that subjects affected by mild depression showed descriptive rather than analytic language style.

Our results also highlighted some features of depressed people's speech, that to the best of our knowledge were not observed yet in other studies. Firstly, depressed participants used fewer words related to the fields of work (for instance words as: “work,” “school,” “working,” “class”) and money (for instance words as: “business,” “pay,” “price,” “market”; see Figure 4); this may be related to the fact that one of the major psychological symptoms of depression is apathy, which is characterized by the lack of interest in different aspects of life, including normal daily and social activities.

It was also observed that depressed participants used more words related to the LIWC categories Physical (words as: “medic,” “food,” “patients,” “eye”) and Feeling (words as: “feeling,” “feel,” “hard,” “cool,” “felt”; see Figure 5); specifically, the fact that depressed people use more words that refer to the perceptual sphere could be explained by depressed people's tendency in exhibiting an elevated self-focused attention (Watkins and Teasdale, 2004) which then translates into a tendency in paying more attention on their internal physiological states and perceptions, and therefore talk more about them.

A similar study (Ilias et al., 2023) using linguistic analysis showed that individuals who are under stress and/or depression are more likely to employ terms from particular LIWC categories. More specifically, people with depression tend to focus on the present without making plans for the future, they often talk about negative topics such as death, illness, mental health, and substances, tend to use more swear words and their posts on social media are filled with sadness, anxiety, and a negative tone.

With regard to cross-cultural differences in the speech content of participants suffering from depression, our results highlighted that Russian depressed subjects showed lower verbal production (indicated by a low total Word Count) compared to English depressed participants from Northern Ireland. Concerning the latter (English depressed subjects) this group showed a specific difference compared to Italian and Russian depressed participants consisting in the tendency of using fewer negations (“not,” “no,” “never,” “nothing”) while speaking. Interestingly, the group that showed more differences in the speech content when compared with the others was the group of Italian depressed subjects, which, when compared with Russian and English depressed subjects, showed a decreased use of total pronouns (“I,” “you,” “that,” “it”), 2nd and 3rd person personal pronouns (“you” and “they”), prepositions (“to,” “of,” “in,” “for”) and words related to work (“work,” “school,” “working,” “class”) and money (“business,” “pay,” “price,” “market”; see Figures 6, 7). These results appear to be pretty innovative considering the scarcity of studies investigating how cultural differences affect the linguistic characteristics of depression (De Choudhury et al., 2017; Loveys et al., 2018), and the fact that our study is the first, at least to the best of our knowledge, comparing depressed participants belonging to three European areas: Western Europe (Northern Ireland), Southern Europe (Italy) and Eastern Europe (Russia). Two interesting points emerge: First, among the compared European cultures, Southern European individuals with depression showed a less informal language compared to those from Western and Eastern Europe. This is expressed, for example, in the reduced use of pronouns by Italian participants compared to English and Russian participants (as highlighted by Jordan et al., 2019). The second interesting result concerns the fact that depressed subjects belonging to Southern Europe, compared to those from Western and Eastern Europe, used fewer words relating to the work and financial spheres. This result may reflect two aspects, on the one hand a cultural tendency which is reflected in giving less space to these issues as they could be considered non-fundamental, on the other hand, however, work and money are issues that significantly influence people, determining their lifestyle. Consequently, as previously mentioned, the lack of focus and interest in these aspects could be linked to the apathy caused by depressive disorders.

The observed differences and similarities among participants from Northern Ireland, Italy, and Russia offer interesting insights to understand the potential influence of cultural specificities on the linguistic manifestation of depression. Regarding the differences in pronoun usage, our results highlighted that Italian depressed participants tended to use a lower number of total pronouns, second-person, and third-person pronouns, compared to English and Russian participants. This trend could reflect the communicative style typical of Italian culture, which is often characterized by a high level of contextualization (Triandis, 2018). In high-context cultures, such as Italy, a significant portion of the message is conveyed through context, interpersonal relationships, and nonverbal communication, reducing the need for explicit pronoun use. Conversely, individualistic cultures, such as England, tend to favor a more direct and self-oriented communication style (Hofstede, 2001). The more frequent use of pronouns in English participants may reflect this cultural emphasis on individual expression. Another interesting difference concerns the use of negations. We observed that English depressed participants used fewer negations compared to Italian and Russian participants. This finding could be interpreted in light of the different cultural ways of expressing uncertainty or disagreement. While some cultures may prioritize a more direct and assertive expression, others may adopt a more nuanced or indirect communicative style. The English communicative style, while generally direct, may include mitigation mechanisms that result in a less frequent use of explicit negations. Furthermore, it is interesting to note that Italian depressed participants showed fewer words related to work and money compared to participants in the other two groups. This tendency may reflect a different cultural emphasis attributed to these areas of life. In some cultures, work and economic success may play a central role in individual identity and well-being, while in others they may be considered less of a priority compared to other values, such as family relationships or emotional well-being. It is possible that this different cultural emphasis is reflected in the relative importance that people place on these topics in their discourse. The similarities observed across the groups, despite cultural differences, are also noteworthy. For example, the increased use of first-person singular pronouns (“I”) in depressed participants was found in all three groups. This result suggests that some linguistic characteristics of depression may transcend cultural differences, reflecting fundamental psychological processes common to all individuals experiencing this condition. It is crucial to emphasize that these interpretations are hypothetical in nature and require further investigation.

## 5 Limits of the study

The results may have been affected by confounding factors, such as participants' age, education level and occupation. The educational level of the participants may influence the complexity and structure of the language used. Participants with a higher level of education may use more articulate and formal language, while those with a lower level of education may prefer simpler and more direct language. Participants' occupation may also influence their use of language, particularly in terms related to work and finance. Professionals in specific fields may use technical and industry-specific language, while others may prefer more general language. Unfortunately, data regarding participants' educational level and occupation were not available. However, it is possible to deepen the effect of participants' age. Russian participants, being significantly younger than English and Italian participants, tend to use more informal and direct language. The younger age of Russian participants may explain their more informal language use, as indicated by their longer average sentence lengths. This could indicate a more verbose and less structured use of language, which can be interpreted as a more informal form of language. It was also noticed that Russian participants used more second-person personal pronouns (“you”) than Italians. The frequent use of personal pronouns may be an indicator of more direct and informal language. In addition, Russian participants used more negatives (“not,” “no,” “never,” “nothing”) than English participants, reflecting a more colloquial and less formal tone. These confounding factors may have a significant impact on the results of our analysis. Although we have discussed the effect of age, further studies should also consider educational level and occupation to provide a more complete understanding of the variables that influence language use.

Moreover, while our study provides valuable insights into cross-cultural differences in the linguistic expression of depression, it is important to acknowledge the limitation of not employing within-language standardization. As demonstrated by Dudău and Sava (2021), language structure can significantly influence LIWC-22 outputs. These structural differences encompass variations in grammar, vocabulary, and semantics. Consequently, some of the cross-cultural variations observed may reflect differences in language rather than pure differences in the linguistic manifestation of depression. Future research should address this limitation by exploring and applying appropriate within-language standardization techniques to better isolate the effects of culture and depression, potentially by employing language-specific control groups or statistical methods designed to account for cross-linguistic variance. Moreover, in interpreting the cross-cultural comparisons, it is important to acknowledge that the English LIWC-22 has a stronger base of validation research compared to the Italian and Russian LIWC-22 dictionaries. Future research could contribute to the field by further investigating the psychometric properties of LIWC-22 in diverse languages.

## 6 Conclusions

The novelty of the described work is first of all represented by the focus on verbal behavior analysis as a methodology to detect depression from people's linguistic content and style, an approach that differs from the one widely used in recent years based on the analysis of acoustic features, such as pitch, prosody, loudness, rate of speech, etc. (He and Cao, 2018; Vázquez-Romero and Gallardo-Antolín, 2020; Wang et al., 2021; Kim et al., 2023). Moreover, our results point toward an interesting observation that could potentially contribute to our understanding of depression from a linguistic standpoint. We observed that participants in the depressed group appeared to speak with less self-regulation and perhaps more spontaneously compared to healthy subjects, an effect that, to the best of our knowledge, has not been extensively documented in previous literature. However, this observation is preliminary and requires further research to confirm its validity and explore its implications.

It could be hypothesized that this tendency of depressed subjects is linked to depressive symptoms which cause lack of interest in what concerns the sphere of sociality, and therefore consequently subjects tended to express themselves with little self-censorship during interaction, possibly due to a lack of interest and commitment to presenting a socially acceptable image. This is only one possible explanation for the observed phenomenon and this aspect needs to be further investigated. Another innovation is represented by the use of Whisper, a totally new tool for the automatic transcription of audio data.

Hopefully, data collected through the present study will be useful in providing guidelines for the design of autonomous systems able to detect early signs of mood changes and depression through the analysis of interactional exchanges. The involvement of different cultures within the study enhances the significance of our findings in relation to machine-driven assessments of depression, especially in clinical contexts, since addressing misclassification is crucial for accurate diagnosis and treatment and recognizing and mitigating cultural biases can enhance the validity of research and ensure that assessments are equitable across diverse populations. This approach can ultimately contribute to better mental health outcomes. The final aim is to provide automated and cost-effective technological interventions to be used in health care centers, as well as by mental health professionals, such as psychologists, psychiatrists, psychotherapists, therefore with the aim to provide assistance, jointly with the clinician's expertise, in the process of diagnosing depression.

## Data Availability

The raw data supporting the conclusions of this article will be made available by the authors, without undue reservation.
